# Sleep in Older Adults and Its Possible Relations With COVID-19

**DOI:** 10.3389/fnagi.2021.647875

**Published:** 2021-06-11

**Authors:** Gabriel Natan Pires, Isabela Antunes Ishikura, Sandra Doria Xavier, Caetano Petrella, Ronaldo Delmonte Piovezan, Ellen Maria Sampaio Xerfan, Monica Levy Andersen, Sergio Tufik

**Affiliations:** ^1^Departamento de Psicobiologia, Universidade Federal de São Paulo, São Paulo, Brazil; ^2^Department of Otolaryngology, Santa Casa de São Paulo, São Paulo, Brazil; ^3^Programa de Pós-Graduação em Medicina Translacional, Universidade Federal de São Paulo, São Paulo, Brazil

**Keywords:** sleep, 2019-nCoV, COVID-19, elderly, corona virus, novel corona virus, SARS-CoV-2, sleep deprivation

## Abstract

Since the beginning of the COVID-19 pandemic, older adults have been found to be a highly vulnerable group, with a higher prevalence of severe cases and negative outcomes. Research has focused on the reasons why older adults are at greater risk; Sleep-related factors have been suggested as one possible explanation for this. An individual’s sleep pattern undergoes significant changes over the course of their life. In older adults a specific sleep profile can be observed, one characterized by advanced sleep timing, a morningness preference, longer sleep-onset latency, shorter overall sleep duration, increased sleep fragmentation, reduced slow-wave sleep and, increased wake time after sleep onset. Additionally, an increased prevalence of sleep disorders can be observed, such as obstructive sleep apnea and insomnia. Previous research has already linked sleep disorders (especially sleep apnea) with COVID-19, but few studies have focused specifically on the older population. We believe that the intrinsic sleep patterns of older adults, and the prevalence of sleep disorders in this population, may be important factors that could explain why they are at a greater risk of negative COVID-19 outcomes. In this review, we discuss the relationship between sleep and COVID-19 among older adults, focusing on three different aspects: (1) Sleep-related issues that might increase the likelihood of getting infected by SARS-COV-2; (2) Sleep disturbances that might increase the predisposition to worse COVID-19 prognosis and outcomes; and (3) COVID-19-related aspects affecting community-dwelling older adults, such as social isolation, quarantine, and home confinement, among others, that might impact sleep.

## Introduction

Since the beginning of the COVID-19 pandemic, older adults have been one of the most vulnerable groups, with a higher prevalence of severe cases and negative outcomes [such as intensive care unit (ICU) admissions and death; Guan et al., 2020; Zhou et al., 2020]. Due to this close and clear relationship, research has focused on the reasons why older adults are at greater risk.

**Figure 1:**
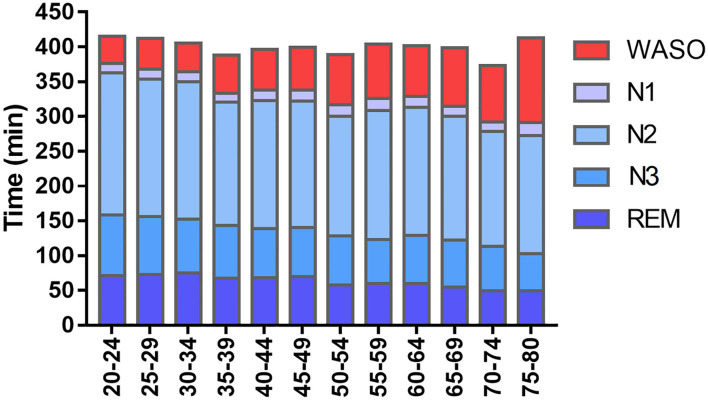
Sleep structure throughout the lifespan. Values obtained in relation to prevalence obtained by the São Paulo Epidemiologic Sleep Study. Comparing the 20–24 to the 75–80 age strata, reductions in total sleep time (376–291 min), N3 sleep (87–53 min) and REM sleep duration (70–49 min), and an increase in time awake after sleep onset (WASO: 39–141 min) can be observed. A reduction in sleep efficiency (87–64%) and an increase in sleep latency (14–35 min) were also observed (data not shown). The reductions in N3 and REM sleep should be contextualized in respect of the global reduction in total sleep time. In terms of percentage, REM sleep proportion stays stable (18–17%) while slow-wave sleep is subjected to a small reduction (24–18%). Adapted from Moraes et al. (2014).

Among the age-related changes observed in multiple physiological functions and behaviors, sleep pattern across lifespan undergoes marked modifications and a specific sleep profile can be defined for each major life stage. Total sleep time, sleep efficiency, and the proportion of sleep changes from the first weeks of life until old age (Roffwarg et al., 1966; Moraes et al., 2014; Boulos et al., 2019; as shown in Figure 1). Changes can also be seen in other aspects of sleep, including sleep microarchitecture, circadian preferences, and the prevalence and clinical presentation of sleep disorders.

The relationship between sleep disorders and COVID-19 has already been discussed but has mostly focused on the general population (Agoramoorthy et al., 2020; Altena et al., 2020; De Mello et al., 2020; Jahrami et al., 2020; Meira E Cruz et al., 2020; Morin et al., 2020; Roitblat et al., 2021). There has been less discussion about the specific sleep patterns and prevalence of sleep disorders in older adults, which might mediate the increased severity of COVID-19 in this population.

In this review, we discuss the relationship between sleep and COVID-19 in older adults. We start with a brief overview of the sleep characteristics and sleep disorders in older adults, and how aging changes the relationship between sleep and immunity. We then examine how sleep and COVID-19 are associated in older adults, focusing on three different aspects: (1) sleep-related factors that increase the likelihood of being infected; (2) predisposing elements that worsen prognosis; and (3) COVID-19-related aspects, such as social isolation, home confinement, and quarantine, among others, and how they might impact sleep in older adults.

## Sleep in Older Adults

Aging is characterized by significant physiological alterations in the human body. Studies with older adults and centenarians have revealed the essential role of sleep in respect of longevity (Mazzotti et al., 2014). Sleep is widely recognized to be an elementary physiological process for good health that supports vital restorative functions. More specifically, the maintenance of healthy sleep habits is associated with successful aging, a term used to describe older people who have no significant detriment in their physiological, physical, and social functioning, and no major disease (Rowe and Kahn, 1997). Conversely, sleep disturbances in older adults are associated with impairment in these domains.

**Figure 2:**
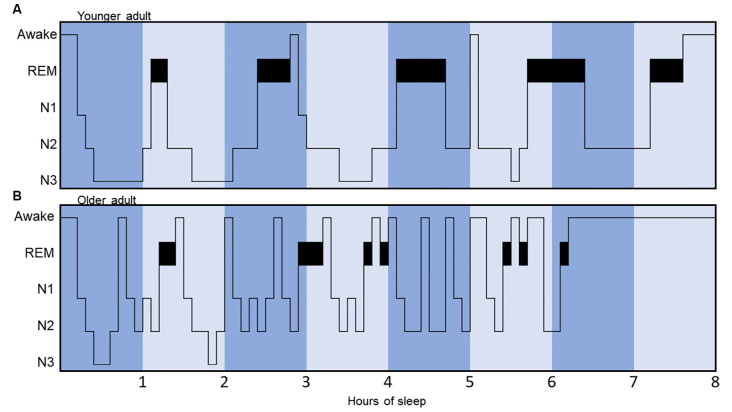
Hypnograms illustrating and comparing sleep architecture between a younger and an older adult. The younger adult hypnogram **(A)** displays a regular cyclicity, with appropriate total sleep time (8 h), five sleep cycles, few arousals, more N3 sleep (slow-wave sleep) concentrated in the first half of the night and more REM sleep concentrated in the second half of the night. Conversely, the older adult hypnogram **(B)** displays a more fragmented sleep pattern, with less clearly identifiable sleep cycles, less N3 sleep, reduced total sleep time, and early awakening.

The main alterations in sleep observed in older adults include altered sleep architecture, reduced total sleep time, a preference for morningness, and reduced slow-wave activity (Figure 2). Additionally, comorbidities associated with aging, such as chronic pain, nocturia, diabetes, anxiety, and depression provoke awakenings during sleep reducing the efficiency and the amount of sleep (Foley et al., 2007; Vitiello, 2009).

In young adults, the average total sleep time is 6.5–8.5 h per night, but this is reduced to 5–7 h per night in older adults (Ohayon et al., 2004; Mazzotti et al., 2014). At 60 years of age, a further decline in sleep efficiency can be observed (Ohayon et al., 2004; Mazzotti et al., 2014). Studies have revealed a negative correlation between short sleep duration and successful aging (Liu et al., 2016; Foscolou et al., 2019). A recent Chinese study found a higher prevalence of successful aging in participants sleeping 7 h, and a lower prevalence in participants sleeping <6 h more than four times per week (Liu et al., 2016).

The high incidence of awakenings during aging may contribute significantly to reducing sleep efficiency and increasing daytime naps, as 25% of older adults reported daytime sleepiness severe enough to impair daytime functioning on a regular basis (Foley et al., 2007). A study with Mediterranean older adults observed that midday nappers were more physically active and presented a 20% higher successful aging index score (Foscolou et al., 2019). Midday napping has more pronounced effects in adults aged >80 years, with a nine-fold increase in the odds of having a high successful aging score (Foscolou et al., 2019). On the other hand, excessive diurnal sleepiness has been associated with an increased risk of adverse outcomes in older adults, including lower gait speed and disabilities (Nakakubo et al., 2016; Tyagi et al., 2017). These findings reveal the restorative function sleep may have in the older population.

A number of studies (Ohayon et al., 2004; Foley et al., 2007; Vitiello, 2009) showed that good sleep is an important factor in respect of functioning in a variety of domains that impact the quality of life of older adults. Sleep plays a critical role in cognitive function, as confirmed by impaired psychomotor vigilance, reaction time, memory and, learning following a sleep restriction protocol (Lim and Dinges, 2010). Poor sleep in adults >75 years old predicts future declines in mental and physical adaptation, fewer social activities, greater depressive symptoms, and more chronic medical burden (Dew et al., 2003). The risk of mortality increases 1.9-fold in older adults with a sleep efficiency of <80% (Dew et al., 2003), and short sleep duration has been shown to be a predictor for cardiovascular diseases (Gangwisch et al., 2006; Hoevenaar-Blom et al., 2011; Faraut et al., 2012), obesity, diabetes, and stress (Tufik et al., 2009; Grandner et al., 2016). Alongside behavioral factors, such as diet, physical exercise, smoking, and social life, sleep plays an important role in successful aging.

## Sleep Disorders and Complaints in Older Adults

### Obstructive Sleep Apnea

Obstructive sleep apnea (OSA) is the most common type of sleep-disordered breathing (SDB) in the general population, and in the older population (Young et al., 2002; Tufik et al., 2010). It is characterized by the reduction (hypopneas) or complete cessation (apneas) of airflow in the upper airways during the night (Kimoff et al., 1994; Patil et al., 2007).

**Figure 3:**
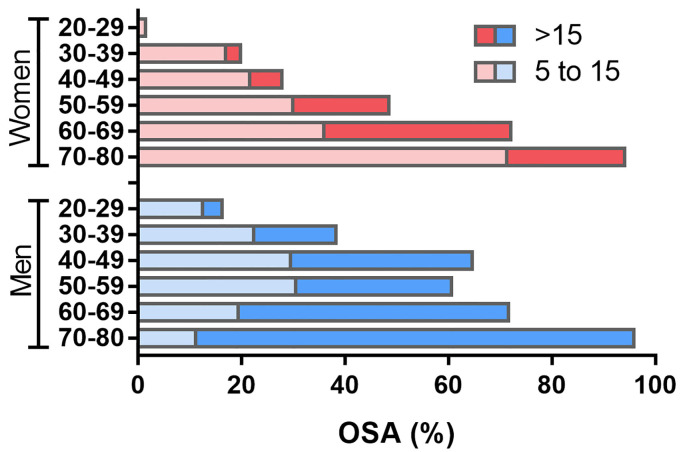
Prevalence of obstructive sleep apnea (OSA) from 20–80 years old. Data are presented for each 20 years range, separately for men and women. Apnea-Hypopnea Index (AHI) ranging from 5–15 represents mild OSA, while an AHI>15 represents moderate to severe OSA. Considering the older adult population, the prevalence of OSA is remarkably high, but the proportion of moderate to severe OSA is higher among men, while mild OSA is more common among women. Values refer to the prevalence obtained in the São Paulo Epidemiologic Sleep Study. Adapted from Tufik et al. (2010).

Different studies have estimated that OSA incidence ranges from 5.6% to 60% in people >65 years (Ahmad et al., 2014; Suzuki et al., 2017), with age being positively related with an increase in its prevalence (Morrell et al., 2012; McMillan and Morrell, 2016; Yaremchuk, 2018). A population-based survey (Tufik et al., 2010) showed a prevalence of OSA of 32.8% in the general population, with a progressive increase with aging, reaching 60.2% in those aged 60–69 years and 86.9% in those aged 70–80 years (Figure 3). Another population-based study comprising 5,615 men and women between 40–98 years of age found that OSA is most frequent in subjects aged 60 years or older—approximately 50% had an apnea and hypopnea index (AHI) of 5–14, and approximately 20% had an AHI ≥15 (Heinzer et al., 2015, 2018). The main cause of the high prevalence of OSA among older adults is increased pharyngeal collapsibility (Bixler et al., 1998, 2001; Vicini et al., 2018; Posadas et al., 2020).

OSA among older adults is not so closely related with excessive daytime sleepiness as it is among younger adults (Whitney et al., 1998; Bixler et al., 2005) and the harmful consequences of OSA are usually milder in older patients than in younger ones. It has been proposed that this protection against OSA cardiovascular consequences among older adults are due to mild cycles of hypoxia-reoxygenation leading to ischemic preconditioning, inducing favorable vascular remodeling (Lavie and Lavie, 2006). On the other hand, the presence of OSA in older adults is associated with clinical and neuropsychiatric manifestations not frequently observed in younger adults; such as dementia, depressive symptoms, epileptic crises, glaucoma, unexplained nocturia, frequent falls, and cardiovascular events (Lévy et al., 1996; Collop, 1997; Launois et al., 2007), as well as more recently described geriatric syndromes, such as frailty, sarcopenia, and sarcopenic obesity (Piovezan et al., 2015, 2019). SDB in older adults is associated with reduced quality of life (Stepnowsky et al., 2000; Baldwin et al., 2001; Kezirian et al., 2009), increased low-grade systemic inflammation (Chung et al., 2009) and cardiovascular alterations (McMillan and Morrell, 2016). Neurocognitive repercussions of OSA have also been observed, as Alzheimer’s disease patients have a five times higher chance of having OSA than cognitively non-impaired individuals of similar age (Emamian et al., 2016). In addition, OSA treatment may decelerate dementia progression in early Alzheimer’s (Ancoli-Israel et al., 2008; Cooke et al., 2009; Troussière et al., 2014).

Continuous positive airway pressure (CPAP) is the gold standard treatment for OSA (Nurwidya et al., 2016; Weiss and Kryger, 2016), and improves several OSA consequences (including daytime sleepiness, quality of life, and metabolic, cardiovascular, and neurocognitive parameters) and has proved to be an effective treatment among older adults (Troussière et al., 2014; Cao et al., 2017; Ponce et al., 2019; Richards et al., 2019).

### Insomnia

Insomnia is characterized by complaints relating to: (1) initiating sleep; (2) maintaining sleep throughout the night; or (3) early awakenings and inability to return to sleep. To be diagnosed as insomnia, there need to be daytime symptoms and it must happen even when sleep opportunity and the environment for sleeping is adequate (AASM, 2014).

A meta-analysis of the prevalence of insomnia in the general population estimated it to be 22.0% (Zeng et al., 2020). The actual prevalence in older adults is hard to estimate, as there are few population-based studies that have diagnosed insomnia in samples of older adults. The prevalence of insomnia is subjected to high variability among studies, due to methodological inconsistencies and different diagnostic criteria. A population-based study in the city of Sao Paulo found that the prevalence of clinical insomnia peaks between 30 and 39 years old, reaching 34.7%, but reducing to 16.8% in people 50–59 years old and to 9.1% in individuals above 60 years old (Castro et al., 2013). The same study showed that the prevalence of insomnia symptoms is reasonably stable throughout the whole lifespan, ranging from 18.4 to 21.5% in the general population and reaching 19.6% above 60 years old (Castro et al., 2013). Another population-based study, representing the population of South Korea, found the prevalence of clinical insomnia (based on national records of hypnotic drugs prescription and use of ICD-10 codes compatible with insomnia) to be 10.3% in people over 60 years old and increasing to 18.2% in people over 80 years old (Chung et al., 2020). In Canada, a nationwide representative survey estimated the prevalence of self-reported insomnia symptoms among people over 65 years old to be 22.2% (Chaput et al., 2018). Other studies, mainly based on clinical samples or on non-standard diagnostic methods, have observed a prevalence of insomnia as high as 60% overall (Kamel and Gammack, 2006), 51% in Denizli, Turkey (Korkmaz Aslan et al., 2020), 44.5% in Chongqing, China (Zou et al., 2019), 32.8% in Osan-Si, South Korea (Kim et al., 2017) and 30.7% in Cuenca, Spain (Redondo-Martínez et al., 2000).

The causes of insomnia in older adults are multifactorial, but usually include changes in daily routine, underlying medical conditions, behavioral aspects, and environmental conditions. Important factors are the incidence of acute medical illnesses, hospitalization, changes in the sleep environment, medications, and psychosocial stress (Kamel and Gammack, 2006).

### Other Sleep Disorders

Other sleep disorders also have specific patterns in older adults. The prevalence of sleep-related movement disorders usually increases with age, including restless legs syndrome (RLS) and periodic limb movement disorder (PLMD; Yaremchuk, 2018). The prevalence of PLMD is of about 45% among older adults, compared to 6% in young individuals (Yaremchuk, 2018). Conversely, the prevalence of sleep bruxism (which is a movement disorder according to the International Classification of Sleep Disorders - 3rd Ed.) was reportedly reduced among older adults (Manfredini et al., 2013), although a population-based study in São Paulo, Brazil found it to be stable throughout life, ranging from 6.3% to 11.3% and reaching 9.2% in those older than 60 years old (Maluly et al., 2013).

REM sleep behavior disorder (RBD) is a REM parasomnia closely related to advanced age. It is more common in men, with initial symptoms being more likely to be observed in the 6th decade of life (Olson et al., 2000; Suzuki et al., 2017). It is characterized by the maintenance of muscle tonus during normal REM sleep (called REM without atonia) resulting in patients acting out their dreams, which are usually of an aggressive nature (Högl et al., 2018). This disorder has an increased prevalence in older individuals and can coexist or precede the diagnosis of neurogenerative diseases by many years, including Parkinson’s disease and Lewy body dementia, is currently considered as an early or prodromal manifestation of the typical motor and cognitive symptoms of these conditions (Dauvilliers et al., 2018; Högl et al., 2018).

### Sleep Deprivation

Sleep deprivation is a generic term that encompasses different types of sleep loss, including extended wakefulness (total absence of sleep for shorter periods), sleep restriction (chronic reductions in total sleep time per night) and sleep fragmentation (recurrent awakenings or arousals throughout the night). Sleep deprivation is one of the major problems of modern society and it has health, social, and economic implications (Foster and Wulff, 2005; Malik and Kaplan, 2005).

Although sleep deprivation and insufficient sleep syndrome are more commonly associated with adolescent and younger adults (AASM, 2014), often as a result of work- or lifestyle-related factors of modern societies (e.g., shift-work, excessive workload, parenthood, social jetlag, nighttime leisure activities, among others; Komada et al., 2008; Chattu et al., 2018), older adults are not fully protected from it. The normal sleep profile of older adults includes increased sleep fragmentation and more awakenings, consequently resulting in increased daytime sleepiness and daytime naps (Mander et al., 2017).

Older adults have been considered to be resistant to sleep deprivation, as they have less sleep rebound, subjective sleepiness, and attention deficit following sleep deprivation and restriction protocols (Münch et al., 2004; Adam et al., 2006; Dijk et al., 2010; Mander et al., 2017). That does not mean that the sleep need or sleep pressure in older adults is reduced, but rather that their sleep-generating capacity is impaired (Mander et al., 2017). Sleep deprivation in older adults should be contextualized to their age range. According to the Sleep Health Foundation, the recommended total sleep time for older adults is between 7 and 8 h; sleeping 5 and 6 h or around 9 h might be appropriate, and sleeping less than 5 h or more than 9 h is considered to be not appropriate (Hirshkowitz et al., 2015). In a sample of 3,576 older adults from Spain, 9.8% of them reported sleeping less than 5 h (López-García et al., 2008), while in a sample of 4,064 older adults from Taiwan, 26.2% reported sleeping 5 h or less (Chen et al., 2013).

## Sleep and COVID-19 Among Older Adults

It is possible that the specific sleep patterns found among older adults may underlie their increased susceptibility to COVID-19 and the severity of the disease. Three general scenarios are possible in this context: (1) sleep-related issues might increase the likelihood of getting infected by SARS-COV-2; (2) sleep disturbances might increase the predisposition to worse COVID-19 prognosis and outcomes; and (3) COVID-19-related aspects especially affecting community-dwelling older adults, such as social isolation, quarantine, and home confinement, among others, may impact sleep.

A selection of important sleep disorders and associated conditions among older adults is listed below, and their possible relationship with COVID-19 is detailed.

### Obstructive Sleep Apnea

Many studies have related OSA with COVID-19, especially as severe cases of both conditions have similar profiles: they are worse among people who are obese, male, aged over 60, and with cardiometabolic dysfunction (Miller and Cappuccio, 2020; Tufik, 2020; Tufik et al., 2020). The fact that older adults have a significantly higher prevalence of OSA might contribute to the increased likelihood of negative outcomes in this population, mostly by predisposing patients to the incident and worsened hypoxemic states and cardiovascular events that increase the odds of negative respiratory and cardiac COVID-19 outcomes (De Mello et al., 2020).

This relationship has been confirmed by large observational studies with clinical samples. A study of 4,668 individuals with positive COVID-19 RNA PCR results has shown that the mortality rate is higher among OSA patients (11.7%) compared with controls (6.9%; Cade et al., 2020). Significant, but smaller associations with OSA were also observed with negative COVID-19 outcomes (ICU admission, mechanical ventilation, and death) and with inpatient admission (Cade et al., 2020). Another study with 9,405 COVID-19 patients found that a history of OSA was more common among those requiring hospitalization (15.3% vs. 3.4% among those who did not) and among those who presented respiratory failure (19.4% vs. 4.5% among those who did not; Maas et al., 2020). A recent meta-analysis has corroborated the fact that OSA increases the risk of hospitalization among COVID-19 cases (Strausz et al., 2021).

The possible mechanisms for this relationship are still under discussion. A provisional explanation involves the onset of cardiovascular events caused by OSA, mainly arrythmias, which have been related to COVID-19 complications. Tachycardia is a primary effect of the increased sympathetic tonus observed in OSA patients and intermittent hypoxia might be an underlying factor for both atrial and ventricular arrythmias (Di Fusco et al., 2020). An increased inflammatory pattern caused by OSA, particularly among obese patients, might also contribute to this relationship, as it might worsen hypoxemia in these patients (Miller and Cappuccio, 2020).

Information about other viral respiratory diseases reinforces the relationship between OSA and COVID-19. Untreated OSA patients have 4.7-fold higher odds of hospitalization from influenza infections (Mok et al., 2020). Sleep apnea (both obstructive and central) has been found to be an independent risk factor for ICU admission among influenza patients (Beumer et al., 2019). The prevalence of sleep apnea was 11% among influenza patients requiring ICU admissions, while among influenza patients in regular wards it was 3% (Beumer et al., 2019). OSA is also a risk factor for lower airway infections and pneumonia, mostly due to upper airway dysfunction (Chiner et al., 2016), which is more common among older adults.

Regarding the treatment of sleep apnea, it is possible that adherent CPAP users are at lower risk of severe cases (Mutti et al., 2020), although further investigation is warranted. It is not clear whether untreated OSA patients diagnosed with COVID-19 would benefit from CPAP, as the cardiovascular and inflammatory background caused by years of untreated OSA would hardly remit in the short term. It has been recommended that the initiation of CPAP therapy should be restricted to severe cases, as the risk of aerosolization and possible infection of people in the same area outweighs the benefits of the treatment in mild to moderate cases (De Mello et al., 2020). In these cases, myofunctional, physical and, respiratory therapies have been suggested, as some can be delivered via telemedicine (De Mello et al., 2020), being also more target-oriented to the pathophysiology of OSA in older adults.

### Insomnia

The incidence of insomnia symptoms, insomnia disorders, and other subjective sleep complaints during the pandemic is likely to increase, probably in all age groups. A meta-analysis comprising 55 studies and a sample of 189,159 individuals indicated a 23.87% prevalence of insomnia during the pandemic (regardless of the diagnosis of COVID-19; Cénat et al., 2021). In another meta-analysis considering 31 studies and 5,153 individuals, the pooled prevalence of sleep disturbances was 34% among COVID-19 patients (Deng et al., 2021). A few studies have compared the prevalence of insomnia before and during the pandemic. Two studies have demonstrated that the prevalence of insomnia has increased by about 7% during the pandemic (Lin et al., 2021; Sun et al., 2021). However, the quality of the current evidence is low, as most studies are simple cross-sectional surveys on insomnia symptoms or retrospective studies subjected to recall bias (Morin and Carrier, 2021).

There is only limited data available regarding older adults. In a sample of 7,127 adults >50 years old from London, 37.1% reported having poor sleep at least once a week during the pandemic, while 17.0% reported having it at least three times a week (Robb et al., 2020). In a Swedish study that evaluated 1,854 individuals aged 70 and older, 23.5% reported having poor sleep due to the pandemic (Gustavsson and Beckman, 2020). In China, a study with a sample of 583 individuals >60 years old reported that the prevalence of moderate to severe insomnia had slightly increased, from 10.8% before the pandemic to 11.9% during it. A meta-analysis concluded that higher age is associated with a higher prevalence of sleep problems during the COVID-19 pandemic, both among the general population and among COVID-19 patients (Jahrami et al., 2020).

Several circumstances resulting from the COVID-19 pandemic might act as precipitating factors for insomnia, especially among older adults, including social isolation, home confinement, anxiety, fear of getting infected, stress, and economic uncertainties (De Mello et al., 2020; Xue et al., 2020). Being alone is probably the most important factor, and some studies have already demonstrated that the subjective feeling of loneliness became more prevalent and more associated with insomnia during the pandemic (Parlapani et al., 2020; Wong et al., 2020; Grossman et al., 2021). Although living alone is an important determinant of the subjective feelings of loneliness in older adults, it is also impacted by related instances, such as poor family functioning and poor social support (Wong et al., 2020). In a sample of 583 older adults from China, the prevalence of moderate to severe loneliness increased from 40.6% to 70.4% (although only 14.3% of them were living alone; Wong et al., 2020). Coping strategies and resilience mediate this relationship, as the impact of loneliness on sleep problems tends to be higher among older adults with lower resilience scores or more COVID-19-related worries (Grossman et al., 2021).

The pandemic and all its related circumstances (home confinement, social distancing, feelings of loneliness, etc.) may act as insomnia precipitators and perpetuators, meaning that they can both trigger insomnia and make it chronic. Given the possible long-term effects of the pandemic on mental health (da Silva et al., 2020), it is possible that some individuals will present persistent insomnia even when the pandemic is over. This is especially true for the older population, due to the levels of loneliness and home confinement in these cases.

Telemedicine was becoming increasingly common in sleep medicine even before the pandemic (Zia and Fields, 2016), but due to the social isolation recommendations, telemedicine and different types of remote medical treatment have been preconized (Hollander and Carr, 2020). Cognitive behavioral therapy for insomnia (CBTi), which is the first line of insomnia treatment and can also improve other subjective sleep disturbance parameters (Morin et al., 2015), has been adapted to online delivery in three different methods: telemedicine (regular CBTi program with psychologists in a virtual environment), internet-based platforms and mobile phone applications. The last two options have been proven to be as effective as standard CBTi, with results sustained up to 1 year after finishing the program (Seyffert et al., 2016; Ye et al., 2016; Zachariae et al., 2016). However, older individuals may have problems to adhere to internet-delivered treatments. In a study about the feasibility of using sleep-related mobile applications (including a sleep diary and behavioral interventions) during the COVID-19 lockdown in France, older adults failed to complete the screening overview more often and found the app-based approach less credible than younger ones (Philip et al., 2020).

Sleep hygiene is an important set of guidance, practices, and behaviors that can help to promote healthy sleep habits and prevent the onset of insomnia symptoms. There is no standard list of recommendations, but the World Sleep Association has recently released what they have called “the Ten Commandments of Sleep Hygiene for Adults (available at https://worldsleepday.org/10-commandments-of-sleep-hygiene-for-adults). These items are valid for older adults and recommendations, such as keeping a regular sleep-wake schedule, being active during the daytime, having some sun exposure, practicing physical activity, and avoiding light-emitting devices close to bedtime are especially relevant (De Mello et al., 2020; Erren and Lewis, 2020). A broader list of sleep hygiene advice tailored to the older population and contextualized to the COVID-19 pandemic is presented in Box 1.

Box 1 **Advice for healthy sleep during the pandemic.**

Below is some advice to prevent circadian misalignment, sleep deprivation, and insomnia symptoms due to lockdown, social isolation, or home confinement for the older population [based on Erren and Lewis (2020), Barone et al. (2020), and Morin et al. (2020)].

– *Regular sleep-wake schedule*: regular awake and bedtimes should be maintained, including at weekends.

– *Regular daytime activities*: daytime activities such as meals and housework should be part of a regular schedule and should ideally be kept at the same time every day, as a way to entrain the circadian rhythm based on social cues. If possible, meals and other activities should be done with the relatives who live together.

– *Regular physical activity*: ideally in the morning or early afternoon, and prescribed by a physical education professional to assure proper adaptations to the home environment and to avoid injury or accidents.

– *Regular online calls with family and friends*: when social isolation norms do not allow visits, scheduled online calls should be encouraged, ideally at a fixed time. This might help to entrain the circadian rhythm and to prevent feelings of loneliness.

– *Exposure to sunlight in the morning and throughout the day*: even when in home confinement, older adults should be exposed to sunlight as much as possible. Windows and curtains should be kept open whenever possible, especially in the morning.

– *Limited screen time in the evening*: screen light disrupts the physiological pattern of melatonin secretion and the whole circadian timing system. Screen exposure should be avoided at least 1 h before bedtime, especially cell phones and tablets.

– *Limited consumption of caffeinated beverages*: coffee, chocolate drinks, soft drinks, and caffeinated teas should be restricted to the morning or early afternoon.

– *Get out of bed if not able to sleep*: if sleep latency is higher than 15–20 min, get up and return to bed only when sleep is imminent.

– *Avoiding any activity in the bed or bedroom that promotes anxiety*: this includes problem-solving or planning for the next day and watching or reading the news.

The prescription of psychotropic medication for insomnia should be cautiously considered during the pandemic (De Mello et al., 2020). The main risks from a pharmacological standpoint are the interactions of hypnotic medication with drugs used in the treatment of COVID-19, possible liver damage by these medications (given that SARS-CoV-2 already leads to impaired life function) and respiratory depression (Rismanbaf, 2020). Another concern is the possible perpetuation of insomnia or the development of dependence on an otherwise circumstantial insomnia, possibly better managed by cognitive-behavioral interventions. However, these recommendations regarding drug therapy for insomnia are mainly based on opinion pieces and on available pharmacological information, have not been actually tested during the COVID-19 pandemic and are not specifically related to older adults.

### Other Sleep Disorders

Other less prevalent sleep disorders have not received much attention in the still growing literature on sleep and COVID-19, especially in older adults. Yet, a few considerations and conjunctures can be drawn.

A possible increase in the prevalence of sleep bruxism during the pandemic has been suggested, although data are still scarce. This has been corroborated by a single online study conducted with adults in Israel and Poland, in which an aggravation of sleep bruxism symptoms was observed (Emodi-Perlman et al., 2020). The reasons for the increase in bruxism prevalence probably include increased anxiety levels, stress, and other psycho-emotional issues (Almeida-Leite et al., 2020; Emodi-Perlman et al., 2020). It is reasonable to speculate that the same might happen among older adults.

In a study conducted in India with 832 patients with Parkinson’s disease (84% over 50 years old), 135 individuals reported RLS-compatible symptoms and 73% of them (24.7% of the total sample) reported that these symptoms worsened during the pandemic (Kumar et al., 2020). These results are in accordance with a hypothesis that proposed that the stresses and behavioral changes related to the COVID-19 pandemic (including home confinement and social distancing) might worsen or trigger symptoms of RLS (Franco et al., 2020).

In the same study, 147 individuals with Parkinson’s disease indicated having RBD-compatible symptoms (Kumar et al., 2020). Among these, 67 (8% of the total sample) reported that these symptoms worsened during the pandemic. Such a high prevalence is explained by the intimate relationship between RBD and Parkinson’s disease (Mahowald and Schenck, 2013; Högl et al., 2018) and the same should not be expected among non-Parkinsonian older adults.

Data regarding these three disorders should be cautiously analyzed, as they are either indirect evidence from other age groups (as for sleep bruxism) or data based on self-reported symptoms in a sample with a specific background disease (as for RBD and RLS). More studies regarding these disorders in older adults are needed, using better diagnostic criteria and unbiased sample characteristics.

### Sleep Deprivation

Older adults seem to be at particular risk of reducing their total sleep time during the COVID-19 pandemic. In an online study with 843 participants carried out in the United Kingdom, sleep-restricted individuals (those sleeping <6 h per night) were on average older than those not sleep-restricted (Pérez-Carbonell et al., 2020).

Among all the effects of sleep deprivation, the most significant in relation to COVID-19 is the impairment of the immune response. Specific sleep parameters, such as slow-wave sleep intensity (which is reduced in older adults and in cases of OSA and sleep fragmentation), are predictive of the magnitude of antibody response and play a role in the adequate formation of an antigen-specific immune response (Lange et al., 2011). The effects of sleep deprivation on the immunological system are widely understood, and it is now recognized that lack of sleep affects the integrity of both innate and acquired immunity (Opp and Krueger, 2015; Irwin and Opp, 2017; Besedovsky et al., 2019) leading to immunosuppression and an increase in the risk of viral and opportunistic infections. Indeed, individuals sleeping less than 7 h per night are more susceptible to the common cold (Cohen et al., 2009; Prather et al., 2015). Sleep deprivation around the time of influenza, H1N1, and hepatitis A vaccination prevents or delays antibody production (Spiegel et al., 2002; Lange et al., 2003; Benedict et al., 2012). On average, the response to vaccination in groups with good sleep was double that in sleep-restricted groups (Spiegel et al., 2002; Lange et al., 2003, 2011; Benedict et al., 2012). This response seems to especially depend on proper amounts of N3 sleep, which is associated with high levels of growth hormone (GH), prolactin and aldosterone, and low levels of cortisol; although REM sleep might also have some role (Besedovsky et al., 2019).

These data allow us to speculate that the same could be true in respect of sleep deprivation and COVID-19, including an increased risk of becoming infected, a poorer prognosis, and reduced efficacy of vaccines. The theoretical immunological threats posed by sleep deprivation in the context of COVID-19 have already been discussed, both in general terms (Mônico-Neto et al., 2020; Silva et al., 2020b) and in relation to specific populations (such as shift workers; Silva et al., 2020a). Although no studies have yet been published that evaluate this relationship among older adults, the indirect evidence indicates that the same might be expected in the older population. Follow-up of vaccination cohorts for COVID-19 could provide definitive information regarding the relationship between sleep deprivation and immunization.

Regular sleep-wake schedules should be prioritized and sleep deprivation by any cause should be prevented. Daytime naps are usually not recommended to individuals with insomnia, but sleep-deprived older adults might benefit from napping for up to 20 min around noon, as a way to diminish the deleterious effects of nighttime sleep deprivation (Morin et al., 2020). Naps in the late afternoon should be avoided, as they can reduce sleep pressure and postpone bedtime, thus perpetuating insomnia symptoms and sleep deprivation.

### Circadian Disruption

The pandemic represents an important challenge to the maintenance of regular sleep-wake schedules, a relationship that has already been discussed by some researchers (De Mello et al., 2020; Erren and Lewis, 2020; Morin et al., 2020). Some factors listed as chronodisruptors are mostly related to the adult, economically active population and are not totally applicable to older adults (such as working from home and altered commuting time). However, it does not mean that older adults are at lesser risk of circadian disorders in the current circumstances. The major challenges older adults face to keep a regular sleep-wake schedule during COVID-19 pandemic are described below:

• *Reduced exposure to sunlight*: home confinement and lockdown policies have reduced the opportunity for direct sunlight exposure for most people. This is especially true in respect of older adults, who might have increased their home confinement due to their high-risk condition, which further reduces their sunlight exposure. Natural light is the main driver of human circadian rhythmicity, and chronodisruption is the natural consequence of its lack. Even daylight illuminated rooms or artificial lights do not meet the needs of our circadian timing system, as the intensity and light exposure periods differ from that obtained by natural direct light exposure (Cardinali et al., 2020b).
• *Reduced effect of social zeitgebers*: these are social activities that support the entraining and synchronizing of our sleep-wake cycle. Since the circadian timing system and, more specifically, suprachiasmatic nuclei functionally deteriorates during the aging process (Cardinali et al., 2020b), the role of social zeitgebers is possibly more important among older adults. Outdoors activities at specific times (walking a pet or going to a supermarket/drugstore) and regular social interactions (receiving or visiting relatives, participating in social/religious group meetings) have been impacted by social isolation, resulting in disruptions to circadian rhythmicity and the sleep-wake cycle.
• *Increased screen time*: older adults might have increased their screen time due to social isolation and home confinement. This is more likely to be associated with television use, but older adults are getting increasingly used to computers, tablets and, mobile phones. In comparison with other sources of artificial light (such as incandescent or fluorescent light sources), portable handsets are an important source of blue light, a wavelength spectrum that is observed in regular daylight. Thus, exposure to these light sources effectively suppresses the production of melatonin similarly to the effects of sunlight exposure, potentially promoting circadian misalignment (Chellappa et al., 2011).

A multinational sample of 3,787 individuals from 18 to 60 years old, demonstrated a shift toward eveningness in 66% of the population, especially marked by later bedtimes of at least 1 h (Roitblat et al., 2021). This probably reflects a social jetlag condition that existed prior to the pandemic, but which working from or staying at home has allowed people to adjust their bedtime and awake times to match their personal preferences, rather than to work-related times. This tendency towards eveningness was not observed in older adults, probably because they were less subjected to social jetlag before the pandemic. Conversely, among the individuals classified as having a desynchronized sleep-wake cycle in this same sample (i.e., without a regular sleep-time and wake-up time), 67% were older adults. A link between circadian clock malfunctioning and SARS-CoV-2 infection has already been described (Meira E Cruz et al., 2020), and if true, would be a possible explanation for the higher likelihood of infection among older adults. In any case, this relationship is mostly based on theoretical assumptions, and more studies are needed regarding chronodisruption and COVID-19 in older adults.

Melatonin is a hormone synthesized mainly by the pineal gland and also by other nonendocrine organs, including the immune system (Cipolla-Neto and Amaral, 2018; Cardinali et al., 2020b). Melatonin has been discussed as a potential therapeutic agent for COVID-19 (Cardinali et al., 2020a,b; Miller and Cappuccio, 2020; Obeysekare et al., 2020; Ramlall et al., 2020), which would potentially benefit older adults due to their reduced melatonin secretion, as it has demonstrated antiapoptotic, antioxidative, and anti-inflammatory effects (Radogna et al., 2007; Jockers et al., 2016). However, clinical data in relation to this hypothesis is scarce and the available evidence to support the use of melatonin against SARS-CoV-2 has come from *in silico* studies (Artigas et al., 2020), a case series (Hardeland and Tan, 2020), and a preprint retrospective study (Ramlall et al., 2020). A few protocols for randomized controlled trials about melatonin administration for COVID-19 have been registered and their results will be essential in respect of knowledge about the effectiveness of melatonin (Cardinali et al., 2020b; Öztürk et al., 2020).

## Summarizing The Relationship

Given the current evidence and the issues discussed above, there are three possible relationships between sleep and COVID-19 in the older population. These are discussed below.

### Sleep-Related Factors Increasing the Likelihood of Being Infected

Previous experience with other viral respiratory diseases indicates that sleep disturbances might cause immunological imbalances, increasing the likelihood of getting infected. Although this is indirect evidence, it is likely that the same may happen with COVID-19.

Sleep deprivation is the main factor that might impair the immune response, but any type of sleep disorder leading to reduced sleep time or sleep fragmentation might also have similar results. At least two sleep disorders that lead to some sort of sleep disruption might contribute to this relationship: Insomnia, which leads to sleep deprivation *per se*, as total sleep time and sleep efficiency can be considerably reduced, and OSA, which results in sleep fragmentation that might also impair the immunological response and increase the likelihood of becoming infected.

Health professionals dealing with older adults with sleep disorders should be aware of this relationship, in order to promote or increase social distancing and mask usage among their patients, thus reducing the risk of infection and negative outcomes.

### Sleep Disorders Predisposing to a Worse Prognosis

Older adults with pre-existing sleep disorders, when infected, are more likely to present a worse prognosis, including a higher chance of ICU admission, mechanical ventilation, and death. The actual mechanisms responsible for this relationship are not clear, but two possible explanations have been suggested.

The first explanation proposes that there is a mechanistic relationship between OSA and COVID-19. This may happen because sleep disorders promote cardiometabolic and respiratory disturbances (e.g., arrythmias, diabetes, and hypoxia due to SDB), which increase the likelihood of negative COVID-19 outcomes. The inflammatory profile observed in patients with sleep disorders might also contribute to this. This seems particularly likely for older adults with OSA, while there is less evidence for other sleep disorders.

The second explanation proposes that both severe OSA and COVID-19 cases share the same characteristics, including obesity, hypertension and diabetes and are more prevalent among older adults. In this case, OSA does not causally increase the likelihood of a poor COVID-19 prognosis, but individuals with severe OSA are at higher risk due to their previously presented risk factors.

### COVID-19-Related Aspects Impacting Sleep

Social isolation, home confinement, anxiety, fear of getting infected, stress, and economic uncertainties due to the current COVID-19 pandemic directly impact sleep, promoting circadian disruption and acting as precipitators or perpetuators of insomnia. Among older adults, loneliness seems to be an important additional factor. Social and emotional support for this population is important during the pandemic, in order to reduce the emotional burden and, consequently, insomnia and other sleep-related symptoms.

Reduced light exposure, reduced time outdoors, and increased exposure to light-emitting electronics might also contribute to poor sleep quality and alterations in the sleep-wakefulness cycle. Sleep hygiene measures might be a useful alternative to promote better sleep quality, to entrain the sleep-wakefulness cycle, and to prevent insomnia symptoms. For those already facing insomnia symptoms, CBTi is a good treatment option, although in-person appointments might be a challenge due to home confinement and social isolations norms.

## Limitations and Research Agenda

Despite the fact that the relationship between sleep and COVID-19 has been widely discussed in the literature and also in general media outlets, original research on the subject is still scarce. Thus, the discussion of this subject and reviews about sleep and COVID-19 rely on theoretical reasoning and speculation. Indirect evidence is also frequently used, such as when data about other viral respiratory diseases are considered to predict what might happen regarding COVID-19. There are even fewer studies regarding this relationship specifically among older adults. Consequently, data acquired from studies of other age groups are frequently used to predict the possible results among older adults. The use of reasoning, generalizations, assumptions, and indirect evidence is valid, as long as the reader understands its risks. While indirectly predicting the prevalence of a sleep disorder is potentially harmless; suggesting new drugs or treatments that have not yet been tested might bring significant risks.

Even the limited number of original studies regarding sleep and COVID-19 have important limitations. Most are cross-sectional studies, which do not allow the establishment of causal relationships. Some are retrospective, either based on the participant reporting their symptoms during pre-pandemic times (thus being subject to reporting bias) or rely on medical records (which are frequently incomplete or inaccurate). Few longitudinal studies have been performed and even fewer randomized controlled trials. The assessment methods are frequently problematic, as most studies used online research tools, which leads to selection bias, as people with sleep complaints might feel more prone to participate. Very few studies have been performed with polysomnography (none of them specifically focused on older adults).

Thus, future research in this field should focus on longitudinal prospective studies, in order to properly assess the causal relationship between sleep disorders, SARS-CoV-2 infection, and COVID-19 outcomes; hopefully corroborating the current assumptions. Regarding treatment proposals, they should be based on randomized controlled trials.

## Conclusions

The COVID-19 pandemic has been a challenge for the global population, and especially for older adults, who are at a greater risk of complications from this disease. Consequently, factors that might predispose or are somehow related to negative outcomes (such as hospitalization, ICU admission, mechanical ventilation, and death) need to be properly understood and warrant ongoing study. Sleep is potentially related to COVID-19 in many ways, and this relationship seems to be especially relevant in older adults.

It should be mentioned that most of the arguments presented above come from clinical observations, theoretical reasoning, and comparisons with other age groups. Studies about the relationship between sleep and COVID-19 in older adults are still scarce, but those published so far confirm this hypothesis. More studies on the subject are needed in order to confirm these points.

## Author Contributions

GP: project conceptualization, project administration, supervision, writing original draft, and review/editing. II, SX, CP, RP, and EX: project conceptualization, writing original draft, and review/editing. MA and ST: project conceptualization, supervision, and review/editing. All authors contributed to the article and approved the submitted version.

## Conflict of Interest

The authors declare that the research was conducted in the absence of any commercial or financial relationships that could be construed as a potential conflict of interest.

## Abbreviations

AHI: Apnea-Hypopnea Index
CBTi: Cognitive-Behavioral Therapy for Insomnia
COVID-19: Coronavirus Disease 2019
CPAP: Continuous Positive Air Pressure
GH: Growth Hormone
ICU: Intensive Care Unit
NREM: Non-Rapid Eye Movements
OSA: Obstructive Sleep Apnea
PLMD: Periodic Limb Movement Disorder
RBD: REM Behavior Disorder
REM: Rapid Eye Movement
RLS: Restless Legs Syndrome
SARS-CoV-2: Severe Acute Respiratory Syndrome Coronavirus 2
SDB: Sleep-Disordered Breathing
SWA: Slow Wave Activity
WASO: Wake After Sleep Onset.
